# Complete Genome Sequences of Grapevine Red Globe Virus in Japan

**DOI:** 10.1128/mra.00434-22

**Published:** 2022-11-03

**Authors:** Toya Yamamoto, Haruka Sato, Takumi Suzuki, Akio Miyazaki, Yugo Kitazawa, Kensaku Maejima, Shigetou Namba, Yasuyuki Yamaji

**Affiliations:** a Department of Agricultural and Environmental Biology, Graduate School of Agricultural and Life Sciences, The University of Tokyo, Tokyo, Japan; KU Leuven

## Abstract

Two complete and three partial genome sequences of grapevine red globe virus (GRGV) from grapevines (*Vitis* spp.) in Japan were determined.

## ANNOUNCEMENT

Grapevine red globe virus (GRGV) is a tentative member of the genus *Maculavirus* (family *Tymoviridae*) and has a genome consisting of approximately 6,850 nucleotides (nt) of single-stranded positive-sense RNA. GRGV has been reported from at least 14 countries and reported to cause no obvious symptoms on grapevines (*Vitis* spp.) (1–5). Fourteen complete genomes of GRGV have been reported since 2017 (6), but none from Asia. Here, we report the complete genome sequences of GRGV in Japan.

To verify whether commercial grapevine nursery stocks in Japan were infected with GRGV, total RNA was extracted from petioles of 32 stocks as described by Iandolino et al. (7) using 3-mercapto-1,2-propanediol instead of 2-mercaptoethanol and treated with DNase I (Nippon Gene, Japan). Then, reverse transcription-PCR (RT-PCR) was performed with the GRGV-specific primer pair RG6061F and RG6801R (6) using the SuperScript III one-step RT-PCR system (Invitrogen, USA), revealing five GRGV-infected stocks: one Pinot Noir (JP-PN), one Chardonnay (JP-Ch), and three Yama Sauvignon (JP-YS1 to -YS3). Pinot Noir, which was also infected by grapevine leafroll-associated virus 3 (GLRaV-3), grapevine rupestris stem pitting-associated virus, and grapevine fanleaf virus (data not shown), presented a reddish-purple interveinal area and downward rolling of leaves presumably caused by GLRaV-3 (8). Other nursery stocks presented no obvious symptoms.

GRGV genomes were amplified by RT-PCR, using the primer pairs described in Table 1, except for the 5′ ends, which were amplified as described previously (9), and the 3′ ends, which were amplified using LA Taq (TaKaRa, Japan) with GRGV-6678F and GRadaptor-R (Table 1) after reverse transcription with avian myeloblastosis virus (AMV) RTase (Nippon Gene) with the GeneRacer oligo(dT) primer (Invitrogen). The amplicons were directly Sanger sequenced and assembled using ATGC v. 4.3.5 (Genetyx, Japan). Finally, the complete genomes of JP-YS1 and JP-YS3 and partial genomes of JP-YS2, JP-Ch, and JP-PN were obtained.

**TABLE 1:** Primers used in this study

Purpose and primer	Sequence (5′ to 3′)	Amplified region (nt)^*a*^
Detection of GRGV		
RG6061F (14)	CCGAGCTTCTCTCCAAGATCA	6042–6798
RG6801R (14)	ACTTAACGTAGGCCACTGGGT	
5′ RACE^*b*^		
GRGV-290R	GGAATGGACCAGAAGGAATG	
GRGV-351R	GAAGAGGTGGGGGAAGTTGGT	
GRGV-412R	GGAAGTCTCCGAGGAG	
AAP	GGCCACGCGTCGACTAGTACGGGIIGGGIIGGGIIG	
AUAP	GGCCACGCGTCGACTAGTAC	
RT-PCR		
JP-YS1 and JP-YS3		
GRGV-38F	TCTGGTTATCTTGCTCACATTCG	38–1211
GRGV-1211R	CCCCAGGAGTCCAGGATGGAG	
GRGV-970F	TGCCCRACCCTCACCAACC	970–1935
GRGV-1935R	GAACCAGCGGTAGGCCATG	
GRGV-1531F	GCCCACTACTTCCTCTTCCG	1531–3318
GRGV-3318R	GCGCTGGAGCATCTGCTG	
GRGV-3085F	CACAARTACCGMTCCCACC	3085–3659
GRGV-3659R	GACCADAGRCAGTACATGTC	
GRGV-3374F	GGAAGCGCAATCTCAAGATC	3374–6577
GRGV-6577R	GGGGGTGTTGTTGTAGGACAC	
GRGV-5811F	CAAAGCCTTCCGCCCCGAG	5811–6846
GRGV-6846R	GTGGGGGCAAGCAGAGACTG	
JP-PN		
GRGV-2F (6)	TCCGCCCCGAGAAGCACTTTG	5819–6831
RG1980R (15)	GACTGGGAAACTGCACACTACTA	
JP-YS2 and JP-Ch		
GRGV-5866F	CACAACACTGAATCGCTTCC	5866–6797
GRGV-6797R	CTTAACGTAGGCCACTGGGTCC	
Amplification of 3′ ends		
GRGV-6678F	GCTCATCCGTGCGTGTCACC	
GRadaptor-R	CTACGTAACGGCATGACAGTG	
GRadaptor-oligodT	GCTGTCAACGATACGCTACGTAACGGCATGACAGTGTTTTTTTTTTTTTTTTTT	

a The position of amplified fragments based on the complete genome sequence of both JP-YS1 and JP-YS3 (accession numbers LC704876 and LC704878).

b RACE, rapid amplification of cDNA ends.

**FIG 1:**
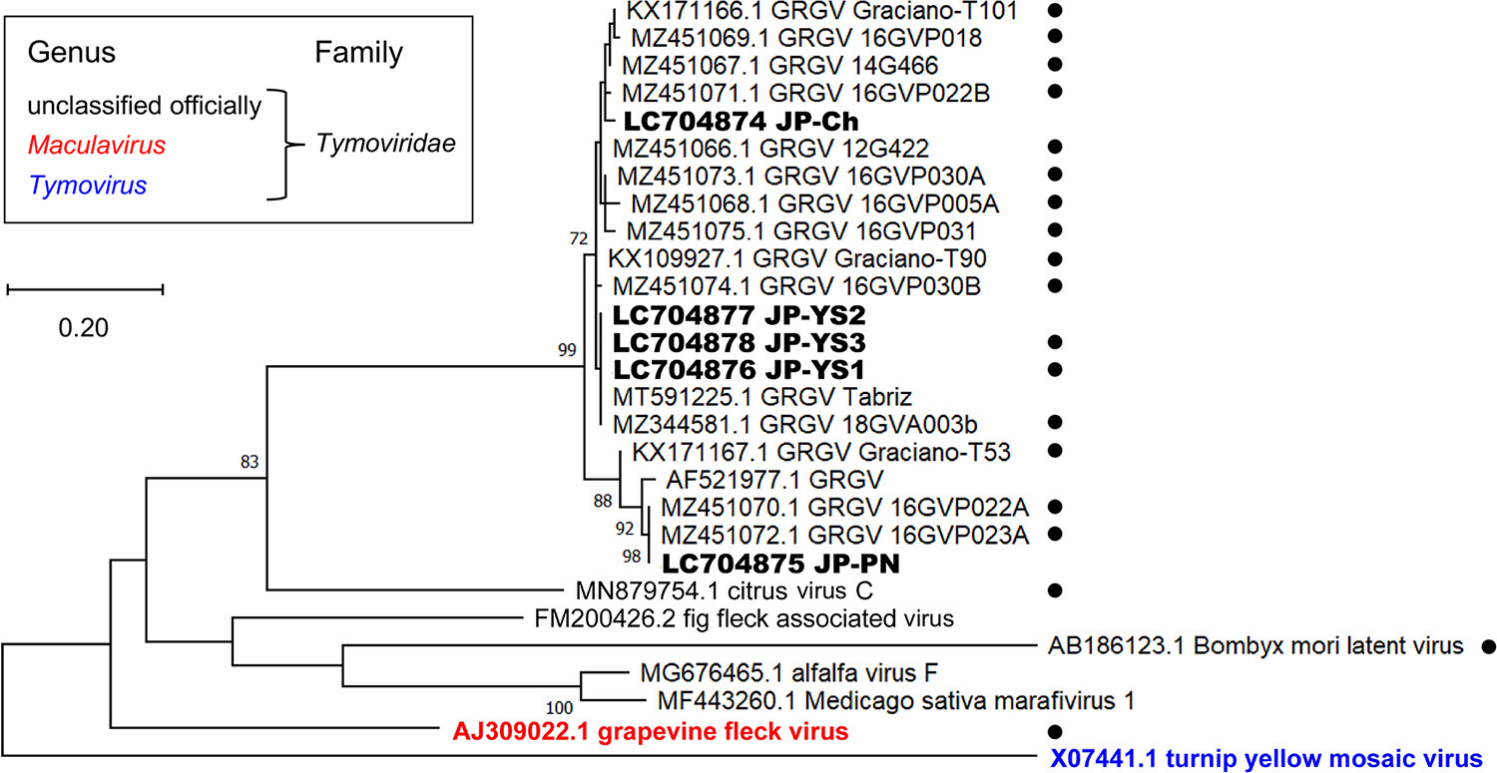
Phylogenetic tree of GRGV and closely related viruses. The tree was constructed based on CP amino acid sequences using the maximum-likelihood method with the LG+G model in MEGA11 (13). Numbers at the nodes are bootstrap values (>70%) obtained for 1,000 replicates. The viruses identified in this study are in bold. The scale bar indicates the number of amino acid substitutions per site. Turnip yellow mosaic virus (genus *Tymovirus*, family *Tymoviridae*) was used as an outgroup. Black dots on the right indicate isolates used to obtain pairwise sequence identities of the complete genome in addition to those of CP.

The complete JP-YS1 and JP-YS3 genomes were both 6,859 nt long, excluding the poly(A) tail. Their structures were identical, and two putative GRGV open reading frames (ORFs) were predicted by NCBI ORF Finder (https://www.ncbi.nlm.nih.gov/orffinder/). ORF1 (nt 199 to 6,132) encoded a putative 220-kDa replicase polyprotein, and ORF2 (nt 6,008 to 6,715) encoded a putative 24.9-kDa coat protein (CP). The partial sequences of JP-YS2, JP-Ch, and JP-PN were also predicted to contain the full ORF2. A phylogenetic analysis of the CP showed that the Japanese viruses formed a monophyletic group with all known GRGVs (Fig. 1).

The sequence-based criteria demarcating species in the genus *Maculavirus* are 80% genome-wide nucleotide identity or 90% CP amino acid identity (10). The pairwise sequence identities of the Japanese isolates with previously reported GRGVs (Fig. 1) were calculated using the MUSCLE algorithm (11) in the program SDT v.1.2 (12). The overall genome sequence identities were 85.5 to 90.8% (JP-YS1) and 85.4 to 90.7% (JP-YS3) at the nucleotide level. Those of the CP were 87.2 to 99.1% (JP-YS1 to -YS3), 87.7 to 98.7% (JP-Ch), and 87.2 to 99.6% (JP-PN) at the amino acid level. Although the amino acid identities of the CP were below the demarcation threshold in some combinations, the high genome-wide nucleotide identity above 80% and the close phylogenetic relationship (Fig. 1) strongly indicate that the Japanese viruses are isolates of GRGV.

### Data availability.

The viral genome sequences have been deposited in the DDBJ under accession numbers LC704874 (JP-Ch), LC704875 (JP-PN), LC704876 (JP-YS1), LC704877 (JP-YS2), and LC704878 (JP-YS3).

## References

[1] Sabanadzovic S, Aboughanem-Sabanadzovic N, Martelli GP. 2017. Grapevine fleck and similar viruses, p 331–349. *In* Meng B, Martelli GP, Golino DA, Fuchs M (ed), Grapevine viruses: molecular biology, diagnostics and management. Springer, New York, NY.

[2] Vončina D, Al Rwahnih M, Rowhani A, Gouran M, Almeida RPP. 2017. Viral diversity in autochthonous Croatian grapevine cultivars. Plant Dis 101:1230–1235. doi:10.1094/PDIS-10-16-1543-RE. 30682947

[3] Czotter N, Molnar J, Szabó E, Demian E, Kontra L, Baksa I, Szittya G, Kocsis L, Deak T, Bisztray G, Tusnady GE, Burgyan J, Varallyay E. 2018. NGS of virus-derived small RNAs as a diagnostic method used to determine viromes of Hungarian vineyards. Front Microbiol 9:122. doi:10.3389/fmicb.2018.00122.

[4] Ruiz-García AB, Nourinejhad Zarghani S, Okic A, Olmos A, Wetzel T. 2018. First report of grapevine red globe virus in grapevine in Germany. Plant Dis 102:1675. doi:10.1094/PDIS-01-18-0105-PDN.

[5] Nourinejhad Zarghani S, Khalili M, Dizadji A, Wetzel T. 2021. First report of grapevine red globe virus in grapevine in Iran. J Plant Pathol 103:661–661. doi:10.1007/s42161-021-00749-w.

[6] Cretazzo E, Velasco L. 2017. High-throughput sequencing allowed the completion of the genome of grapevine red globe virus and revealed recurring co-infection with other tymoviruses in grapevine. Plant Pathol 66:1202–1213. doi:10.1111/ppa.12669.

[7] Iandolino AB, Goes da Silva F, Lim H, Choi H, Williams LE, Cook DR. 2004. High-quality RNA, cDNA, and derived EST libraries from grapevine (*Vitis vinifera* L.). Plant Mol Biol Rep 22:269–278. doi:10.1007/BF02773137.

[8] Burger JT, Maree HJ, Gouveia P, Naidu RA. 2017. Grapevine leafroll-associated virus 3, p 167–195. *In* Meng B, Martelli GP, Golino DA, Fuchs M (ed), Grapevine viruses: molecular biology, diagnostics and management. Springer, New York, NY.

[9] Suzuki T, Iwabuchi N, Tokuda R, Matsumoto O, Yoshida T, Nishikawa M, Maejima K, Namba S, Yamaji Y. 2021. Complete genome sequence of Mirabilis crinkle mosaic virus isolated from pokeweed in Japan. Microbiol Resour Announc 10:e00283-21. doi:10.1128/MRA.00283-21. 34042472 PMC8201631

[10] Dreher TW, Edwards MC, Gibbs AJ, Haenni A-L, Hammond RW, Jupin I, Koenig R, Sabanadzovic S, Martelli GP. 2012. Family *Tymoviridae*, p 944–952. *In* King AMQ, Adams MJ, Carstens EB, Lefkowitz EJ. (ed), Virus taxonomy: ninth report of the International Committee on Taxonomy of Viruses. Elsevier, Amsterdam, The Netherlands.

[11] Edgar RC. 2004. MUSCLE: multiple sequence alignment with high accuracy and high throughput. Nucleic Acids Res 32:1792–1797. doi:10.1093/nar/gkh340. 15034147 PMC390337

[12] Muhire BM, Varsani A, Martin DP. 2014. SDT: a virus classification tool based on pairwise sequence alignment and identity calculation. PLoS One 9:e108277. doi:10.1371/journal.pone.0108277. 25259891 PMC4178126

[13] Tamura K, Stecher G, Kumar S. 2021. MEGA11: Molecular Evolutionary Genetics Analysis version 11. Mol Biol Evol 38:3022–3027. doi:10.1093/molbev/msab120. 33892491 PMC8233496

[14] Jarugula S, Chingandu N, Adiputra J, Bagewadi B, Adegbola RO, Thammina C, Rayapati N. 2020. First report of grapevine red globe virus in grapevines in Washington State. Plant Dis 105:717. doi:10.1094/PDIS-07-20-1609-PDN.

[15] Cretazzo E, Padilla CV, Velasco L. 2017. First report of grapevine red globe virus in grapevine in Spain. Plant Dis 101:264. doi:10.1094/PDIS-06-16-0932-PDN. 30743377

